# Supplementary material for: Changes in Lymphocyte Subpopulations after Remdesivir Therapy for COVID-19: A Brief Report
Source: Int J Mol Sci. 2023 Oct 7;24(19):14973. doi: 10.3390/ijms241914973 (PMC10573452; doi:10.3390/ijms241914973)
Supplement: Supplementary file 1 [file ijms-24-14973-s001.zip › ijms-2622886-supplementary.pdf]

Changes in Lymphocyte Subpopulations after Remdesivir Therapy for COVID-19: A Brief Report

Rossella Cianci, Maria Grazia Massaro, Elisabetta De Santis, Beatrice Totti, Antonio Gasbarrini, Giovanni Gambassi and Vincenzo Giambra

Supplementary Information

including

Supplementary Figure S1

Supplementary Tables S1-S2

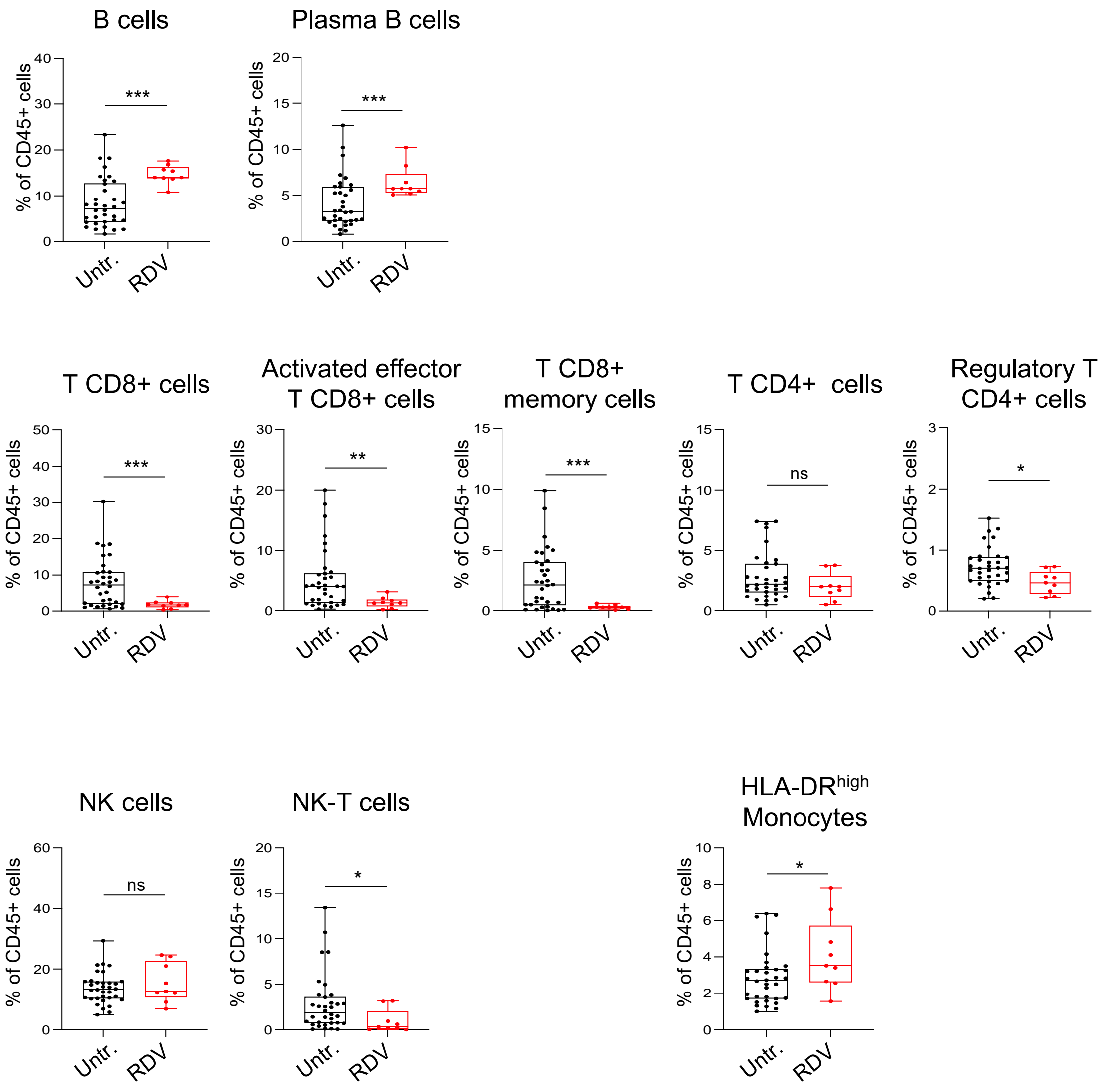

Supplementary Figure S1 related to Table 1. Distribution of lymphocyte subpopulations in patients not in therapy (Untr) or after Remdesivir (RDV) treatment. Welch's t-test statistical analysis was performed on the percentage values of the different fractions of CD45+ cells. *ns, not significant; *, p<0.05; **, p<0.01; ***, p<0.001 (two-tailed unpaired Welch's t-test).*

Laser	Detector	Fluorophore	Marker	Antibody Clone	Company	Catalog #
405	448/59	Super Bright 436	CD3	SK7	eBioscience™	62-0036-41
	525/50	eFluor 506	CD45	HI30	eBioscience™	69-0459-41
	620/29	Qdot 605	HLA-DR	Tü36	eBioscience™	Q10052
	710/45	Super Bright 702	CD127	eBioRDR5	eBioscience™	67-1278-42
	755 LP	Super Bright 780	CD8	53-6.7	eBioscience™	78-0081-82
488	513/26	Alexa Fluor 488	CD19	SJ25C1	eBioscience™	53-0198-42
	795/70	PE-Cyanine7	CD16	eBioCB16 (CB16)	eBioscience™	25-0168-42
561	579/16	PE	CD4	RPA-T4	eBioscience™	12-0049-42
	614/20	PE-eFluor 610	CD56	CMSSB	eBioscience™	61-0567-42
	692/75	PE-Cyanine5	CD38	HIT2	eBioscience™	15-0389-42
640	671/30	APC	CD14	61D3	eBioscience™	17-0149-41
	722/44	Alexa Fluor 700	CD11b	CBRM1/5	eBioscience™	56-0113-42
	795/70	APC-eFluor 780	Live / Dead		eBioscience™	L34975

Supplementary Table S1. Panel of cell surface markers and fluorophore-conjugated antibodies used in the multiparameter flow cytometry assessment of PBMCs. APC, allophycocyanine; PE, phycoerythrin.

	Treated with Remdesivir	
	Pre-Treatment	Post-Treatment
P/F	277 ± 98	304.7 ± 95.1
Venturi Mask Oxygen percentage	0.35 ± 0.12	0.31 ± 0.13
Procalcitonin	0.08 ± 0.06	0.092 ± 0.088
C-reactive protein	78.86 ± 47.9	85.98 ± 78.95
Interleukin (IL)-6 (pg/mL)	8.15 ± 5.3	<2.7± 0
Fibrinogen	584.9 ± 115.8	412.2 ± 108.5
D-Dimer	2980 ± 7216.4	384.1 ± 166.8
Ferritin	989.7 ± 456.8	743.8 ± 503.9
Alanine aminotransferase	41 ± 22.4	68.3 ± 64.7
Aspartate aminotransferase	34.2 ± 19.8	139.6 ± 171.2
Gamma glutamil transpeptidase	61.6 ± 79.8	48 ± 31.6
Alkaline phosphatase	61.8 ± 21.3	55.6 ± 10
Total bilirubin	0.55 ± 0.2	0.43 ± 0.1
Creatinine	0.89 ± 0.15	0.81 ± 0.1

Supplementary Table S2. Clinical features of considered COVID-19 patients before and after Remdesivir (RDV) treatment. Abbreviations: P/F [arterial partial pressure of oxygen (PaO2)/fraction of inspired oxygen (FiO2)].
